# Immunogenicity of a peptide‐based anti‐IgE conjugate vaccine in non‐human primates

**DOI:** 10.1002/iid3.98

**Published:** 2016-04-01

**Authors:** Risini D. Weeratna, Ghania Chikh, Lu Zhang, James D. Fraser, Jennifer M. Thorn, James R. Merson, Michael J. McCluskie, Brian R. Champion, Heather L. Davis

**Affiliations:** ^1^ Pfizer Vaccine ImmunotherapeuticsOttawa LaboratoriesOttawaOntarioCanada; ^2^ Pfizer Vaccine ImmunotherapeuticsLa JollaCaliforniaUSA; ^3^ Pfizer Biotherapeutics Pharmaceutical SciencesSt. LouisMissouriUSA

**Keywords:** Allergy, anti‐IgE antibodies, IgE, IgE peptide conjugates, vaccine

## Abstract

The anti‐human immunoglobulin E (IgE) monoclonal antibody, omalizumab (Xolair®, Genentech, South San Fransisco, CA), is effective in the treatment of poorly controlled moderate to severe allergic asthma and chronic idiopathic urticaria. It acts by specifically binding to the constant domain (Cϵ3) of free human IgE in the blood and interstitial fluid. Although efficacious, use of omalizumab is limited due to restrictions on patient weight and pre‐existing IgE levels, and frequent dosing (q2–4 weeks). A vaccine inducing anti‐IgE antibodies has the potential for similar clinical benefits with less frequent dosing and relatively lower cost of goods. We developed a vaccine containing two IgE peptide‐conjugates targeting the Cϵ3 domain of human IgE. As part of preclinical evaluation of the vaccine to optimize formulation and dose prior to initiating clinical studies, we evaluated the vaccine in non‐human primates, and demonstrate the induction of anti‐peptide antibodies that can bind to conformationally intact human IgE and are capable, at least in some animals, of substantial lowering circulating IgE levels.

## Introduction

The incidence of IgE‐mediated allergic conditions, including allergic asthma and rhinitis, has significantly increased over the last few decades, resulting in significant morbidity and mortality and associated costs 1. In the US, the Center for Disease Control and Prevention reported a 73.9% increase in self‐reported asthma during the period from 1980 to 1996 2, 3, 4.

Despite the availability of multiple treatment modalities, including pharmacotherapy to treat symptoms and immunotherapy to address underlying immunological defects, there remains a significant unmet medical need for new therapeutic approaches to treat and potentially modify allergic diseases 5.

Elevated levels of immunoglobulin E (IgE) are characteristic of allergic diseases 6, 7. Allergen‐specific IgE binds to the high affinity receptor (FcϵRI) found on the surface of mast cells, basophils, and eosinophils; cross‐linking of adjacent bound IgE molecules by environmental allergen leads to the release of histamine and other mediators of allergic disease 8. IgE can also bind to low affinity CD23 receptors on B cells, providing a negative regulatory signal for IgE production 7.

Immunoglobulin E (IgE) has long been used as a diagnostic biomarker, and more recently it has become a therapeutic target. Omalizumab (Xolair®, Genentech) is a humanized anti‐IgE monoclonal antibody that directly targets free IgE, preventing it from binding to FcϵRI. Omalizumab was originally approved for the treatment of moderate to severe allergic asthma in patients who are not well controlled by inhaled corticosteroids 8, 9, 10, 11. It has more recently been approved for the treatment of chronic idiopathic urticaria, which is typically not allergen‐induced but involves IgE nonetheless, at least in some patients 12, 13. Off‐label use of omalizumab, such as for allergic rhinitis and food allergies, has also been evaluated in clinical studies 5, 14, 15, 16. When used at recommended doses, omalizumab results in a rapid, dramatic (>95%), and sustained decline in levels of free serum IgE that is associated with clinical benefit 17. Dosing of omalizumab for allergic asthma is weight based (mg/kg) with the prescribed dose depending on the level of total serum IgE (IU/mL) at initiation of treatment. As such, drug use is limited to patients under a certain weight and/or with baseline IgE under a certain level 18, 19. Prescribing is further restricted due to high cost 20 and frequency of dosing (every 2–4 weeks) and the need for the subcutaneous injection to be performed in the clinic due to rare anaphylactic reactions 21. For chronic idiopathic urticaria, absolute doses of omalizumab are prescribed, but the same limitations exist as for asthma 22.

In theory, an IgE targeting vaccine could provide clinical benefits similar to omalizumab except with less frequent dosing and relatively lower cost of goods 23, 24. It is not desirable to use whole IgE as the vaccine antigen since antibodies that can bind to receptor‐bound IgE could result in cross‐linking and anaphylaxis. A 76‐amino acid peptide corresponding to the region at the border of Cϵ2–Cϵ3 that binds to FcϵRI has been shown to block in vivo passive sensitization of human skin mast cells and in vitro sensitization of human basophil granulocytes with human IgE antibodies 25. A fusion protein consisting of the Cϵ2–Cϵ3 domain of rat IgE fused to carrier protein glutathione‐S‐transferase resulted in IgE lowering in vaccinated rats, as well as reduction in allergen sensitivity 26. While there was no evidence of cross‐linking receptor‐bound IgE in these studies, there remains a potential risk for auto‐reactive T cells with the use of longer peptides. Shorter peptides can reduce this risk, however used alone peptides are poorly immunogenic and, therefore, unlikely to induce the required titers and functionality of anti‐peptide antibodies. The use of a carrier protein to provide structural support of the correct conformation and T‐helper epitopes is required for adequate immunogenicity of self‐peptide based vaccines and for the induction of antibodies that can cross‐react to the intact target molecule 27, 28. One approach is to make recombinant proteins that include the desired peptide sequence, for example, combining the homologous Cϵ3 sequences with heterologous and evolutionary distant Cϵ2 and Cϵ4 domains 29, 30, or cloning the Cϵ3 epitopes into a gene for a foreign protein, such as green fluorescent protein 31. Another approach is to conjugate the peptides to a carrier molecule, as has been demonstrated using one or more Cϵ3‐derived peptides of various lengths conjugated to a carrier such as Keyhole limpet hemocyanin (KLH), surface antigen of the hepatitis B virus (HBsAg), or a purified protein derivative of tuberculin 32, 33, 34. While most studies on anti‐IgE vaccines have been conducted in rodents, IgE lowering following immunization with a peptide conjugate vaccine or a chimeric protein vaccine has also been demonstrated in dogs 34, 35.

A number of IgE peptide antigens were rationally designed, using structural information for IgE and its interaction with the high affinity FcϵRI receptor such that the anti‐IgE antibodies induced by the vaccine will bind free IgE, but not interact with IgE bound to cell surface receptors, thus avoid triggering the release of inflammatory mediators through IgE receptor cross linking 36.

Through extensive screening in mice two peptides were selected for further development. Screening involved immunization of mice with peptides conjugated to a virus‐like particle (VLP) that is derived from Qβ bacteriophage (Qb‐VLP) then ex vivo assessment of anti‐sera by ELISA for high titers of antibodies recognizing human IgE and in vivo functional assessment of rate of clearance of human IgE after bolus administration to mice. Selected peptides were also screened to ensure that induced antibodies do not induce degranulation of FcϵRI receptor‐expressing cells through binding to receptor bound IgE and that they do not induce a T cell responses. The two selected peptides, labeled peptide P and peptide Y, are derived from different loops of the Cϵ3 domain of IgE. Peptide Y corresponds to the epitope recognized by omalizumab while peptide P targets a different loop on IgE Cϵ3 36. Mice immunized with the two conjugates admixed in equal proportions and adjuvanted with aluminum hydroxide (alum) produce high levels of IgE‐specific antibody that can bind to free but not receptor‐bound human IgE and enhances the rate of clearance of human IgE after a bolus injection into immunized mice 36, 37. In rodent species, the human vaccine cannot be used to assess function directly due to lack of homology, however, species‐specific mimetic vaccines containing murine homologues of the Y and P peptides conjugated to Qb‐VLP was shown to be capable of breaking B cell tolerance and induce anti‐IgE antibodies than can lower serum IgE in a murine allergy model 36, 37. Similarly, peptides derived from Cϵ3 domain of canine IgE has been shown to induce anti‐IgE and have therapeutic benefit in multiple dog allergy models 38, 39.

In the current study, we evaluated the anti‐IgE vaccine in non‐human primates (NHP) for immunogenicity and function (lowering of circulating IgE). Cynomolgus monkey (*Macaca* *fascicularis*) was selected since there is a ∼85% sequence homology between human and cynomolgus IgE constant region with a 100% homology for the P peptide and only single amino acid difference for the Y peptide 40.

## Materials and Methods

### IgE peptide: VLP conjugates

Qb‐VLP was made by Pfizer using *Escherichia coli* K12 strain RB791 transfected with the plasmid pTac‐nSDQb‐mut containing the coding region of the Qb bacteriophage protein monomer (NCBI GenBank Acc. No. M99039; nucleotide 46–444). Cells were lysed by homogenization in the presence of Triton X‐100 (Sigma–Aldrich, San Diego, CA). Cell debris was removed by centrifugation. Qb‐VLP was purified using a series of chromatography steps: Fractogel TMAE (EMD Millipore, Darmstadt, Germany), Ceramic Hydroxyapatite Type II (Biorad, Hercules, CA), Phenyl Sepharose (GE Healthcare, Pittsburg, PA), and Sepharose CL‐4B (GE Healthcare). After the final column, the pooled fractions were concentrated to 3 mg/mL using a Pall Ultrafiltration system equipped with a 100 kD Pellicon 2 Biomax V‐screen PES cartridge. Samples were frozen at −80°C until further use.

Peptides P and Y were sourced from Chinese Peptide Company (CPC, Hangzhou, China). The sequence of peptide P is ADSNPRGVSAYLSRPSPGGC and the sequence of peptide Y is QCRVTHPHLPRALMRS. Cysteines were added to each peptide sequence to allow conjugation through the sulfhydryl group of the added cysteine using the bifunctional linker, Succinimidyl‐6‐[ß‐maleimidopropionamido]hexanoate (SMPH; Sigma–Aldrich).

Qb‐VLP conjugates were prepared by a two‐step process which first involved activating the Qb‐VLP with SMPH and secondly, conjugating with peptide. A 10× molar excess (for peptide P) or 4.25× molar excess (for peptide Y) SMPH in DMSO (Sigma–Aldrich) was added to 100 mg of Qb‐VLP at 3 mg/mL in 20 mM sodium phosphate, 150 mM NaCl, pH 7.2. Activation proceeded at 15°C for 5 h with continuous mixing. After 5 h, the activated Qb‐VLP was purified and buffer exchanged into 100 mM sodium phosphate, 300 mM NaCl, pH 6.8 by ultrafiltration/diafiltration (UF/DF) using a Sartorius Slice UF system installed with a Biomax 300 kD membrane. The purified activated Qb‐VLP was diluted to 1 mg/mL with 100 mM sodium phosphate, 300 mM NaCl, pH 6.8, and peptide was added at a 7× molar excess. Conjugation proceeded at 15°C for 1.5 h with continuous mixing. After 1.5 h, the conjugated Qb‐VLP was purified and buffer exchanged into 100 mM sodium phosphate, 200 mM NaCl, pH 7.2 by UF/DF using a Sartorius Slice UF system installed with a Biomax 300 kD membrane. Sucrose at 140 mg/mL and PS20 at 0.2 mg/mL were added to the purified peptide‐Qb‐VLP conjugates. The concentration was adjusted to 2.5 mg/mL and the conjugates were frozen at −80°C until further use.

### Adjuvants

Aluminum hydroxide was obtained in the form of Alhydrogel “85” (Brenntag Biosector, Frederikssund, Denmark) and is hereafter referred to as alum, with doses indicating amount of Al^3+^.

CpG ODN (CpG) (B‐Class with sequence of 5′ TCG TCG TTT TTC GGT GCT TTT 3′) was synthesized by Avecia (Milford, MA) with a nuclease‐resistant phosphorothioate backbone) as described previously 41.

### Animal model and immunization

Cynomolgus monkeys (CiToxLAB., Montreal, QC) (mixed female and male; *n* = 6 to 8/group) aged between 2 and 5 years were used in the studies. Circulating IgE titer in study animals ranged from 10 (lower limit of quantification for the assay) to 3141 U/mL at study start. Animals were randomized into groups with group Geomean titers (GMT) of circulating IgE ranging from 52 to 126 U/mL. Monkeys were immunized as indicated on figure legends with P peptide and/or Y peptide conjugated to Qb‐VLP (P‐Qb and Y‐Qb, respectively) with CpG and/or alum. Doses of antigen and adjuvants are defined in figure legends. All vaccine formulations were made up to a total volume of 1.0 mL with PBS and administered by intramuscular (IM) injection in the left quadriceps muscle. Animals were bled at pre‐determined intervals (weeks 0, 2, 4, 6, 8, 10, 12, 14, 16, 22, 26, and 28) by saphenous vein puncture and plasma/serum used for quantitation of antibody specific to the peptides, whole IgE, or the C3C4 portion of human IgE. In some studies, plasma/serum was also used for quantification of total IgE titer. All procedures performed on animals in this study were in accordance with regulations and guidelines reviewed and approved by the Pfizer Institutional Animal Care and Use Committee and were conducted in facilities fully accredited by AAALAC International.

### Detection of vaccine‐specific antibody titers

Antibody titers against the Y and P peptides, C3C4 domain, or whole human IgE, and the Qb‐VLP carrier were measured by enzyme‐linked immunosorbent assay (ELISA) using plasma or serum samples from individual animals. MaxiSorp 384‐well plates (Fisher Scientific, Ottawa, ON, Canada) coated (12 μL/mL) with whole human IgE (7.5 μg/mL, Abbiotec, San Diego, CA), Qb‐VLP (3 μg/mL), Y or P peptides conjugated to KLH (1 or 2 μg/mL, respectively) were used for the detection of anti‐IgE, anti‐Qb, anti‐Y, and anti‐P titers, respectively. KLH was used for conjugation of peptides used in the ELISA in order to eliminate detection of any anti‐carrier antibodies. Reacti‐Bind™ Streptavidin HBC Clear 384‐Well Plates (Fisher Scientific) coated (12 μL/mL) with biotinylated human C3C4 (1.5 μg/mL, Pfizer, Sandwich, UK) were used to detect anti‐C3C4 titers. Selected samples were similarly tested on plates coated with cynomolgus C2C4 to compare anti‐human and anti‐cynomolgus antibody responses. Coated plates were incubated overnight at 4°C, except for plates coated with human C3C4 or cynomolgus C2C4 which were incubated for 1 h at room temperature. Following incubation, plates were aspirated and washed with PBS (for C3C4 or peptides) or PBS containing 0.05% Tween 20 (for whole IgE or Qb) and blocked 10% bovine calf serum (BCS; Hyclone, Logan, UT) for 1 h at room temperature. Samples were serially diluted in dilution buffer (10% BCS in DPBS) and added to the blocked and washed plates (12 μL/well). Plates were incubated at room temperature for 1 h on a shaker. The plates were washed again with PBS‐0.05% Tween 20 and then incubated with goat anti‐human IgG‐horse radish peroxidase (HRP) (Southern Biotech, Brimingham, AL) diluted with dilution buffer (10% BCS in DPBS) for 1 h at room temperature. The plates were then washed again and incubated with Tetramethylbenzidine (TMB) substrate (Mandel Scientific, Guelph, ON) in the dark for 30 min at room temperature. The reaction was stopped by addition of 25 μL/well of 4N sulfuric acid (Fisher Scientific) and read at 450 nm using automated plate reader. Titers were defined as the highest serum dilution that resulted in an absorbance value (OD 450) two times greater than that of the diluent control. Group values were expressed as GMT ± 95% confidence interval (CI).

Except in cases where anti‐IgE antibodies were detected with various coating reagents (Y, P, C2C4, C3C4, whole IgE) for comparison purposes, C3C4 was selected for routine detection of anti‐IgE since it gave similar titers to those when plates were coated with whole human IgE.

### Quantification of plasma/serum IgE level

The levels of circulating IgE in individual plasma or serum samples were quantified using the Monkey IgE ELISA kit (Alpha Diagnostic International, San Antonio, TX) as per manufacturer's instructions. The sample OD was determined at wavelength 450 nm with OD subtraction at 630 nm using a SPECTRAmax Plus Microplate Spectrophotometer. The pre‐existing endogenous IgE levels were measured in all animals at start of vaccination and the reduction in serum IgE is presented for individual animals as percent change in circulating IgE from baseline.

### Anti‐IgE antibody avidity

Antibody avidity was determined by competition ELISA as previously described 42. In brief, serum or plasma samples previously determined to contain anti‐IgE antibody were diluted in 10% BCS in DPBS to achieve absorbance values of approximately 1.0 at 450 nm. Human IgE (Abbiotec) was serially diluted twofold using the same buffer starting at two‐times the highest desired concentration. Equal volumes of diluted serum/plasma sample or the diluent (10% BCS in DPBS) and IgE were incubated for 1 h at room temperature and then added to human IgE coated plates. HRP‐labeled mouse anti‐human IgG (Southern Biotech) followed by TMB substrate (Mandel Scientific) were used for the detection of antibody binding. OD readings at 450 nm were plotted against the molar concentration of IgE and the 50% inhibition (IC_50_) was extrapolated for each sample tested. Using the competition ELISA whereby IC50 was determined at equilibrium, the IC50 value equates to Kd 43.

### Anti‐IgE antibody specificity (competition)

Antibody specificity was determined using the same method as for avidity with the exception of using additional inhibitors; i.e., human IgG, IgM, IgA (Southern Biotech), and C3C4. For this assay, an equal volume of serially diluted inhibitor and diluted serum/plasma or the diluent (10% BCS in DPBS) were added directly to MaxiSorp 384‐well plates coated with human IgE (7.5 μg/mL, 12 μL/well). The concentration ranges of the inhibitors were from 2.5 to 158 nM. Omalizumab (1 ng/mL, Genentech, South San Francisco, CA) was used as a positive control and the concentration ranges of the inhibitors for Omalizumab were from 0.2 to 13.1 nM. The *K*_d_ value was defined as amount of inhibitor required to achieve 50% inhibition of binding of anti‐IgE antibody present in plasma/serum to human IgE.

### Statistical analysis

Data were analyzed using GraphPad Prism (GraphPad Software, San Diego, CA). Statistical significance of the difference between two groups was calculated by Student's 2‐tailed *t*‐test and between three or more groups by one‐way or two‐way ANOVA followed by post hoc analysis using either Dunn's or Tukey's multiple comparison tests. Differences were considered to be not significant with *p* > 0.05.

## Results

### Immunogenicity of Y‐Qb

**Figure 1:**
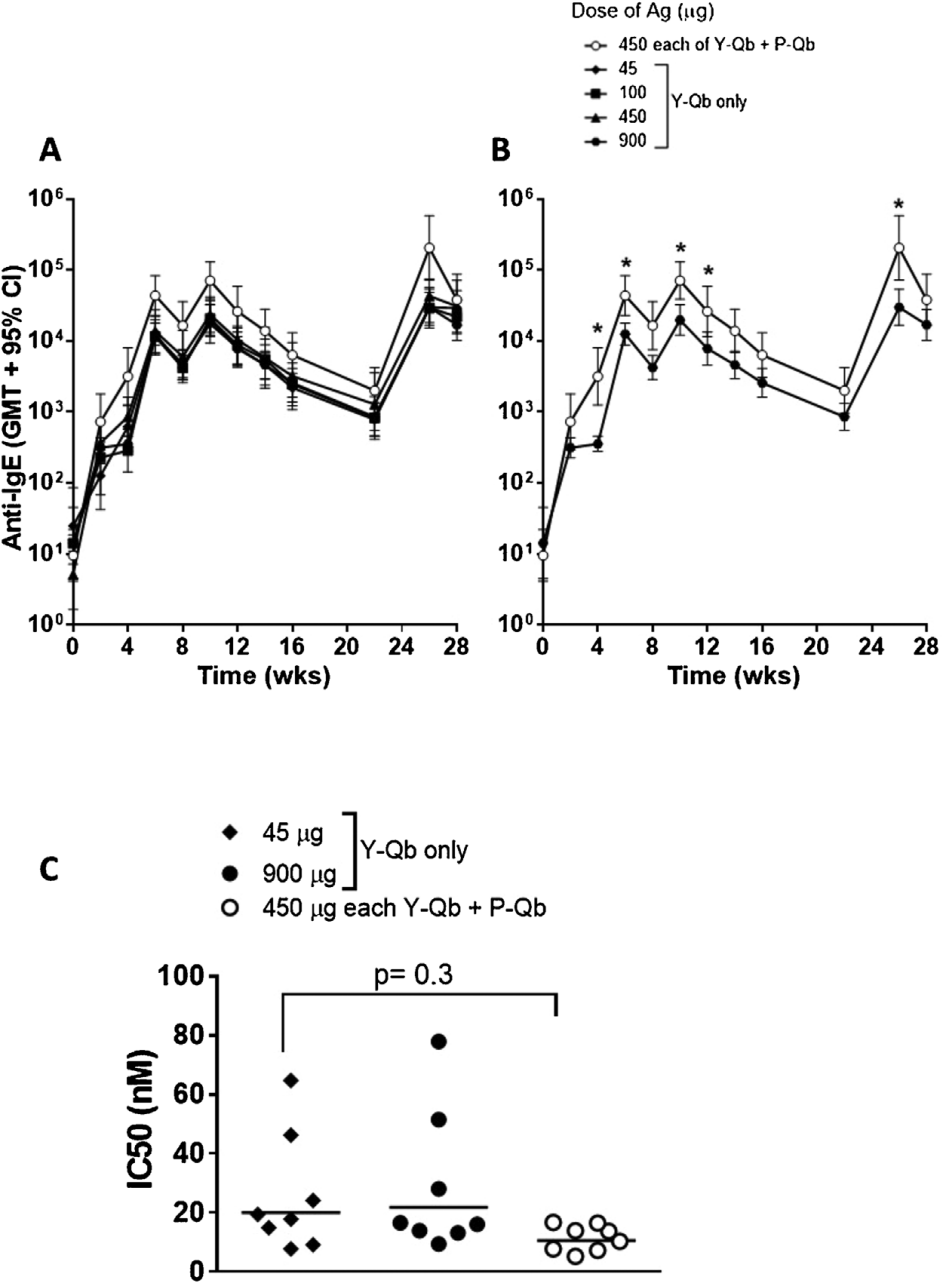
Anti‐C3C4 antibody titer and avidity in monkeys using Y‐Qb alone or Y‐Qb + P‐Qb as antigen. Cynomolgus monkeys (*n* = 8/group) were immunized by IM injection on weeks 0, 4, 8, and 24 with either Y‐Qb alone at 45, 100, 450, or 900 μg or with a mix Y‐Qb and P‐Qb (450 μg each) using alum (800 μg) and CpG (500 μg) as adjuvant. Plasma/serum collected at pre vaccination and at multiple time‐points post immunization was used to determine anti‐IgE antibody titer by ELISA. A: Y‐Qb dose response for induction of anti‐IgE antibody titer. B: Comparison of anti‐IgE titer/kinetics with Y‐Qb alone (900 μg) or a mix of Y‐Qb and P‐Qb (450 μg each). C: Plasma collected at 2 weeks post fourth dose in animals immunized with 45 or 900 μg of Qb‐Y or 450 μg each of Y‐Qb and P‐Qb was used to determine antibody avidity by competition ELISA. Significant differences between groups (*p* < 0.05) indicated by asterisk (*).

Y‐Qb adjuvanted with alum and CpG was tested at different doses in cynomolgus monkeys for immunogenicity and the ability to decrease circulating IgE levels. Using plates coated with human C3C4, it was found that a small number of monkeys (6%) had a low level of pre‐existing anti‐IgE (titers between 100 and 500), however, these pre‐existing titers did not appear to interfere with vaccine immunogenicity and all animals showed an increase in anti‐IgE titers post vaccination relative to baseline (Fig. 1). Only low levels of anti‐IgE titers (<860 GMT at 4 weeks) were observed following the first vaccine administration at all dose levels tested, but these were substantially increased with each vaccine boost. No difference in the kinetics or the magnitude of the anti‐IgE titers were observed between the different antigen dose levels of Y‐Qb tested (Fig. 1A). Similar kinetics and magnitude antibody titers were obtained with selected samples tested on plates coated with human C3C4 and cynomolgus monkey C2C4, with a correlation value of *R* ^2^ = 0.98 (data not shown).

The avidity of antibodies following four administrations of Y‐Qb at 45 and 900 μg (week 26) was tested and there was no antigen dose effect on the quality of the antibody response over this 20‐fold antigen dose range (Fig. 1C).

### Immunogenicity of Y‐Qb + P‐Qb

As shown above, it was not possible to increase anti‐IgE antibody titers with antigen dose. Earlier studies in mice had shown the combination of Y‐Qb and P‐Qb to be superior to Y‐Qb alone for anti‐IgE antibody titer 36, 37. In NHP the Y‐Qb and P‐Qb combination (450 μg each) gave titers that were at least twofold (range 3 to 14‐fold) higher than those induced by an equivalent total dose of Y‐Qb alone (900 μg), and these differences were significant at almost all time points (*p* < 0.05, Fig. 1B). Antibody avidity was not significantly different, but there was a trend toward less inter‐group variability and higher avidity with the Y‐Qb/P‐Qb combination compared to Y‐Qb alone (Fig. 1C). The competition ELISA assay used to measure avidity in this study has relatively low sensitivity, therefore, better differentiation of avidity may have been observed with a more sensitive assay such as surface plasmon resonance using Biocore (GE Healthcare). However, this was not possible due to limitation of samples volume. Furthermore, the assay interference by varying amounts of endogenous IgE within individual monkey plasma may also have impacted the avidity measurement by competition ELISA.

**Figure 2:**
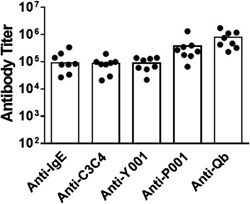
Antibody responses against human IgE and vaccine components in monkeys. Plasma/serum collected at 2 weeks post third dose in the study described in Figure 1 were analyzed for antibody titers against C3C4 domain of human IgE, whole human IgE, Y and P peptides, and Qb VLP by ELISA.

Plasma from animals dosed with Y‐Qb/P‐Qb combination using alum and CpG as adjuvants were tested for antibodies specific to Y and P peptides, C3C4, whole IgE, or the Qb VLP carrier. Animals had pre‐existing antibody titer ≤ 0.25 K GMT against the Y and P peptides, C3C4, whole IgE, or the Qb VLP and titers developed with similar kinetics post immunization. Although these assays are not strictly comparable due to the use of different coating reagents for the sandwich ELISA, similar antibody titers were detected with IgE and C3C4 (GMT of 71 and 90 K, respectively). Therefore, C3C4 protein was used as coating reagent for all other ELISA data reported in this manuscript. Anti‐Y antibody titer (GMT 77K) was also comparable to that for anti‐whole IgE whereas the anti‐P titers were higher (GMT 262K). As expected, high antibody titers developed against the carrier Qb VLP (GMT 633K) (Fig. 2).

**Figure 3:**
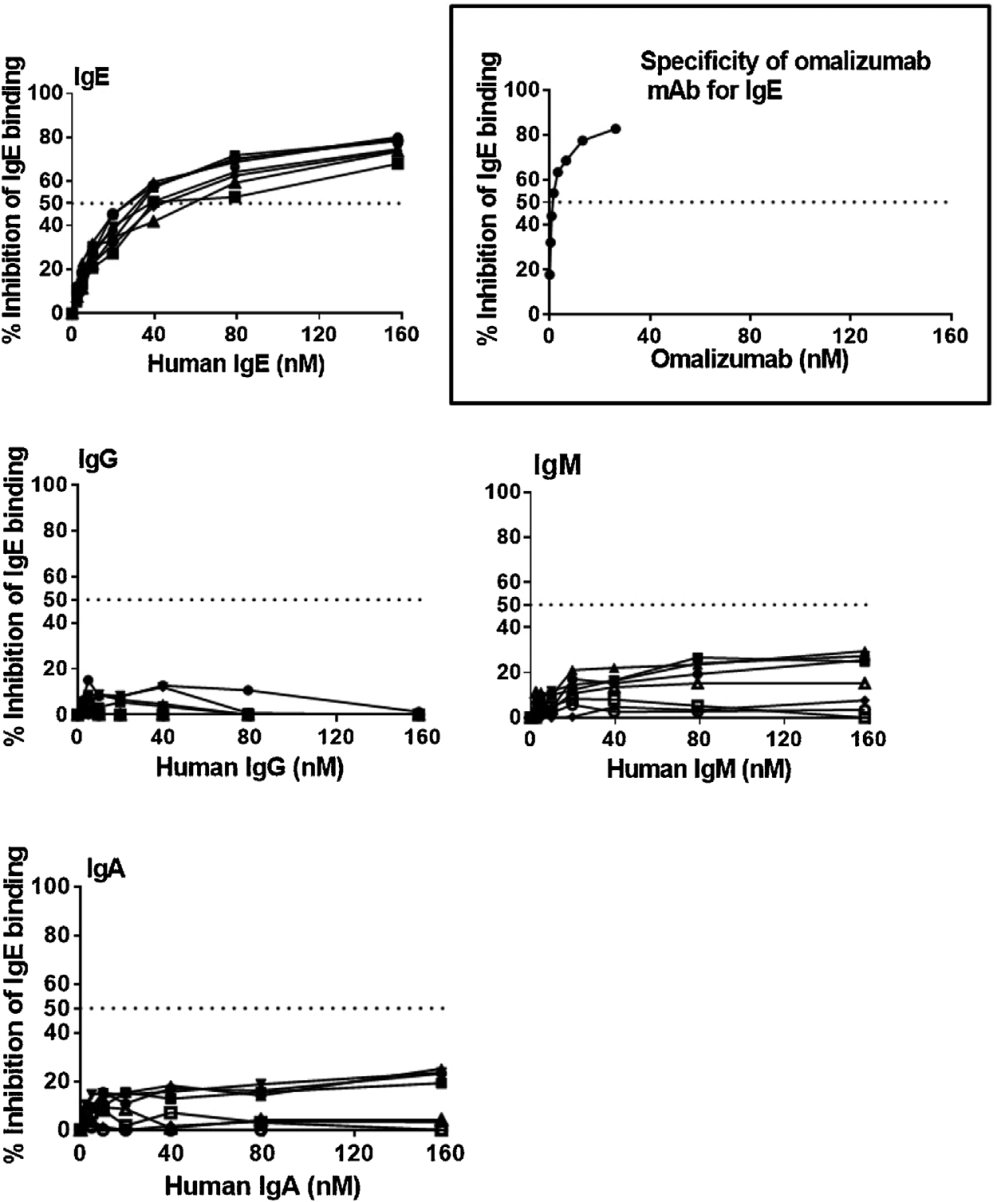
Specificity of vaccine‐induced antibodies. Cynomolgus monkeys (*n* = 8/group) were immunized by IM injection on weeks 0, 4, and 8 with Y‐Qb and P‐Qb at 450 μg each using alum (800 μg) and CpG (500 μg) as adjuvant. Plasma/serum collected at 10 weeks was tested for specificity of vaccine‐induced antibody for binding to human IgE by competition ELISA. Omalizumab was used as positive control. Results presented are the % inhibition in IgE binding.

In a separate study, lower doses of Y‐Qb + P‐Qb (1, 10, or 100 μg each) were tested with alum (250 μg) and CpG (500 μg). Following dosing at 0, 4, and 12 weeks, there was no difference in antibody titer except at 2 weeks after the last dose where 1 µg was inferior (*p* < 0.05) to 10 or 100 μg (GMT = 39, 147, and 110 K, respectively). Anti‐IgE antibodies induced in this study were specific for human IgE and did not cross react with human IgG, IgA, or IgM (Fig. 3). The antigen–antibody dissociation constants (*K*_d_) calculated by ELISA for vaccine‐induced antibody against whole human IgE was 36 ± 11.6 nM whereas that for omalizumab was 0.6 nM using the same assay.

### Vaccine function: reduction in circulating IgE

**Figure 4:**
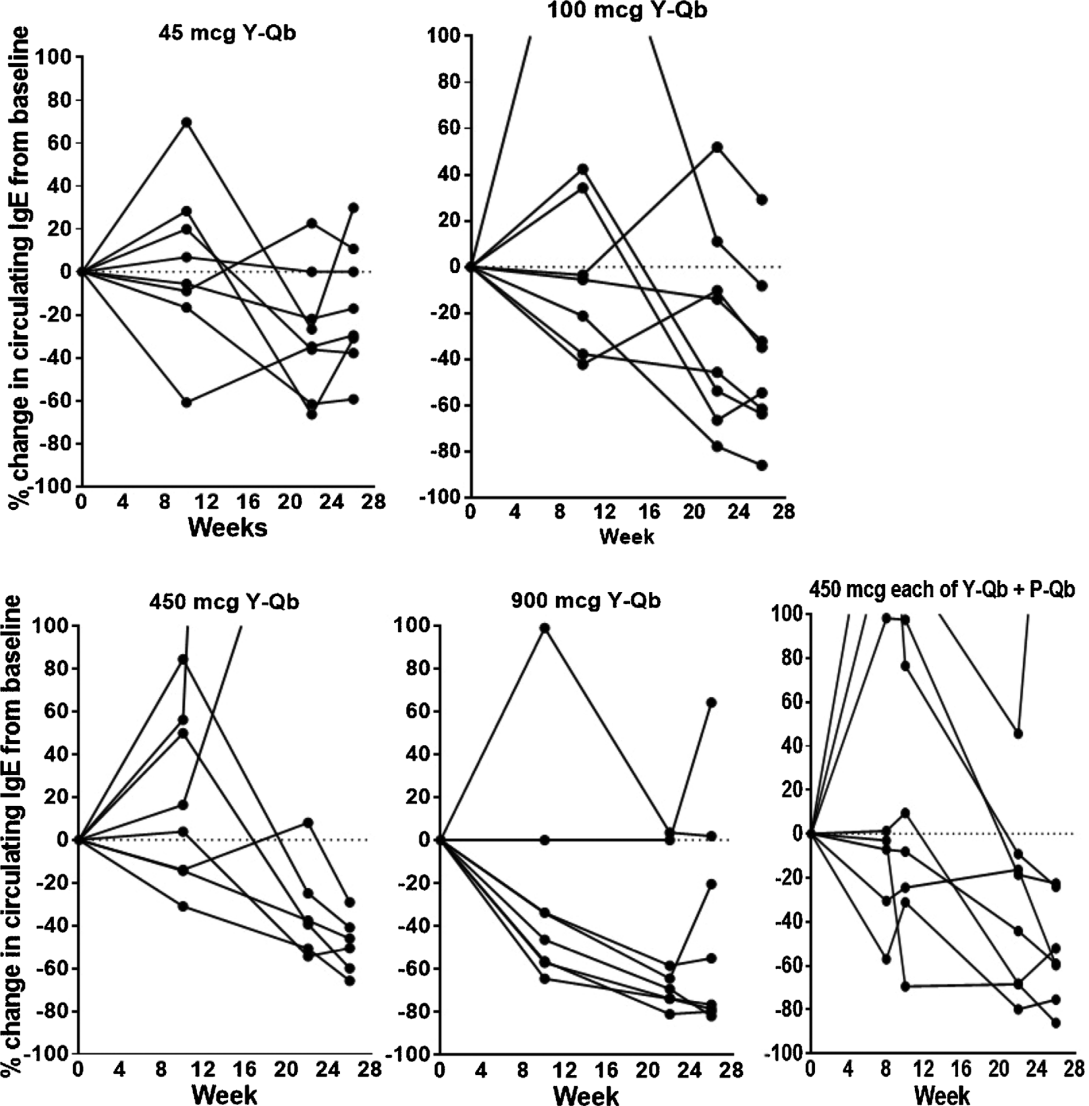
Serum IgE levels in monkeys. Cynomolgus monkeys (*n* = 8/group) were immunized by IM injection on weeks 0, 4, 8, and 24 with either Y‐Qb alone at 45, 100, 450, or 900 μg or with Y‐Qb and P‐Qb at 450 μg each using alum (800 μg) and CpG (500 μg) as adjuvant. Plasma/serum collected at pre vaccination and at multiple time‐points post immunization was used to determine total IgE levels by ELISA. Data are plotted as percent change from the baseline IgE level in the same animal.

Serum IgE levels dropped in some vaccinated animals, with a >50% reduction post fourth dose compared to baseline being achieved in 12.5%, 50%, 50%, and 62.5% of animals in groups receiving 45, 100, 450, or 900 μg Y‐Qb + alum/CpG, respectively. The best kinetics and magnitude of IgE reduction was seen with the highest antigen dose tested (900 μg) (Fig. 4).

Although the antibody titer and avidity appeared to be better with the Y‐Qb/P‐Qb combination, this did not translate into better IgE lowering where Y‐Qb/P‐Qb at 450 μg each was similar to 450 μg Y‐Qb and inferior to 900 μg Y‐Qb as sole antigen (Fig. 4).

It is possible that the presence of endogenous anti‐IgE antibodies may in theory impact the detection of serum IgE levels using the commercial sandwich ELISA kit employed in this study. However, since the vaccine‐induced responses are directed against specific epitopes within the Cϵ3 domain and the diagnostic anti‐IgE reagent is polyclonal, the impact should be minimal. This is confirmed by the fact that in some animals no IgE lowering is detected despite the presence of high levels of anti‐IgE antibodies while in other animals different levels of IgE lowering despite similar magnitude of anti‐IgE titer. There was no correlation between reduction in circulating IgE and anti‐IgE antibody titer or avidity (*r* ^2^ = 0.03), even when analysis was done on a subset of animals with the highest anti‐IgE titers (>5 × 10^4^, *r* ^2^ = 0.07).

### Effect of adjuvants

**Figure 5:**
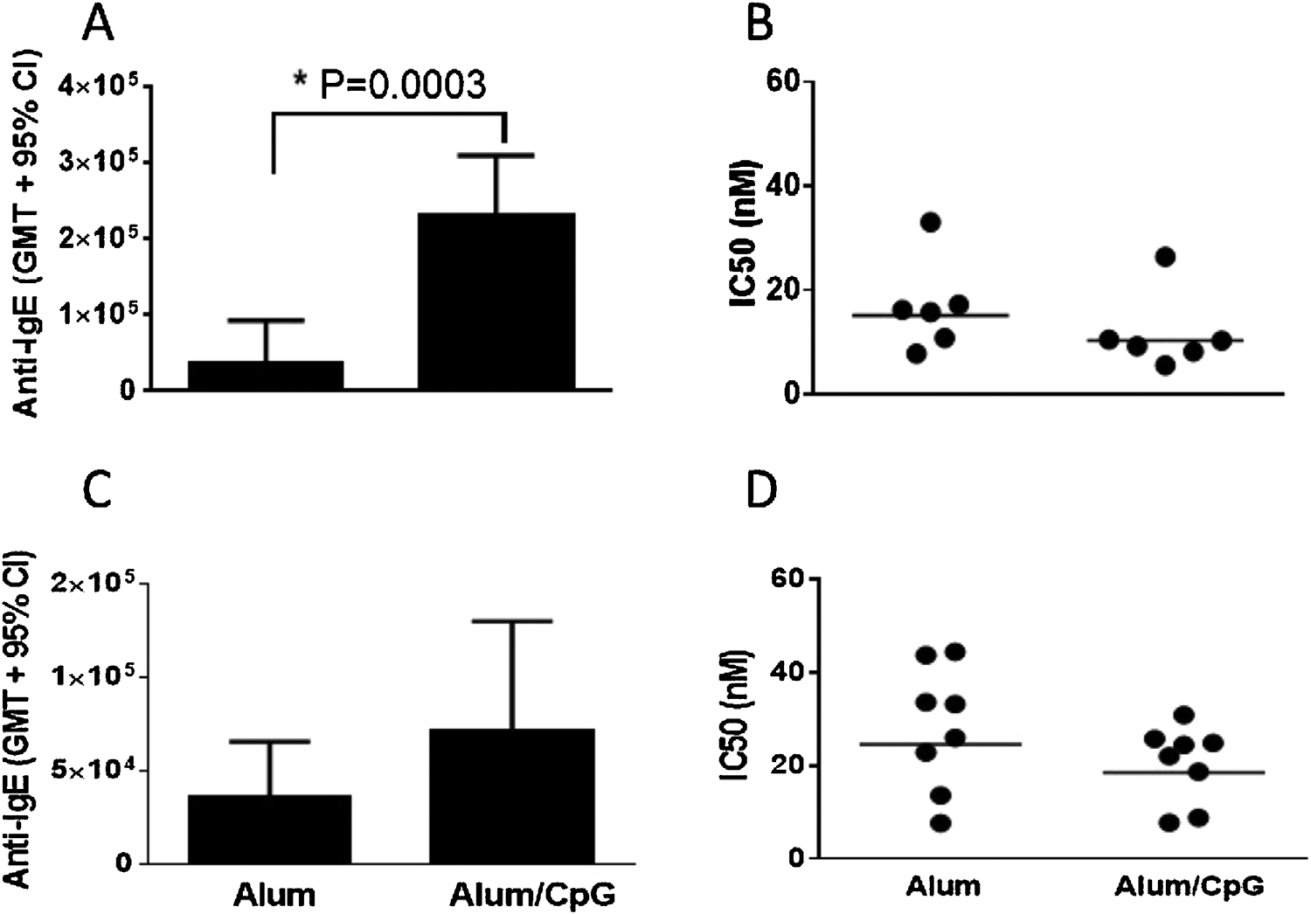
Effect of adjuvants on anti‐C3C4 antibody titer and avidity in monkeys. Cynomolgus monkeys (*n* = 6–8/group) were immunized by IM injection on weeks 0, 4, and 8 with 20 μg (A and B) or 450 μg (C and D) each of Y‐Qb and P‐Qb conjugates using alum (800 μg) with or without CpG (500 μg) as adjuvant. Plasma/serum was collected at 2 weeks post third dose and anti‐IgE titers (A and C) and avidity (B and D) were measured by ELISA.

At a low antigen dose (20 μg of Y‐Qb + P‐Qb combination), CpG inclusion with alum in the adjuvant formulation significantly enhanced the anti‐IgE antibody response compared to alum alone. However, this benefit of CpG was not statistically significant when a high antigen dose (900 μg) was used (Fig. 5A and C, left panel). There was a trend toward greater antibody avidity, with less intra group variability, with alum/CpG compared to alum alone at both antigen doses, albeit with no statistical significance (Fig. 5B and D right panel). Inclusion of CpG with alum had no impact on lowering of circulating IgE (data not shown).

## Discussion

This paper combines the findings from a number of different NHP studies carried out during the preclinical development of this anti‐IgE vaccine.

All vaccine formulations appeared to be well tolerated which is consistent with observations when the adjuvant and carrier components had been tested previously in NHPs and humans at similar or higher doses. Alhydrogel is widely used in licensed human vaccines. The Qb‐VLP has been administered in clinical trials for other indications, for example conjugated to a peptide from angiotensin II for treatment of hypertension (CYT006‐AngQb) 44 or conjugated with a nicotine‐like hapten for smoking cessation (CYT002‐NicQb) 45, 46. The B‐Class CpG ODN used in this study has been tested extensively in NHP during the development of an anti‐nicotine vaccine 42, 47, 48, and is pharmacologically similar to CpG adjuvants that have been tested in phase 1 or 2 studies in combination with alum, as in this study, and as a sole adjuvant in phase 3 studies with the Heplisav™ (Dynavax, Berkley, CA) vaccine against hepatitis B 49, 50, 51, 52, 53, 54.

Peptide screening studies in mice had identified murine homologues of Y‐Qb (mY‐Qb) and P‐Qb (mP‐Qb) as the best peptide conjugates for induction of strong antibody responses that cross‐react with the intact IgE molecule. In mice, both mY‐Qb and mP‐Qb induced similar anti‐IgE responses with the combination being superior to either peptide conjugate alone. However, mY‐Qb was superior to mP‐Qb in lowering serum IgE 36. Since mP‐Qb performed relatively poorly on its own in mice, it was not tested as a sole antigen in this study. In NHP immunized with the two human peptide sequence conjugates together, anti‐P peptide antibody titers were higher than those against anti‐Y peptide, anti‐human C3C4, or anti‐whole human IgE, which were all similar. This suggests that the P peptide is a good immunogen but anti‐P antibodies are less cross‐reactive to intact IgE than anti‐Y antibodies. Similar to that seen in mice, the Y‐Qb and P‐Qb combined vaccine gave higher anti‐IgE antibody titers than the same total dose of Y‐Qb alone. The anti‐IgE antibodies induced by the peptide conjugates were capable of recognizing both human and cynomolgus monkey IgE as shown by similar magnitude and kinetics of anti‐human C3C4 and anti‐monkey C2C3 antibody titers.

There was a mixed antigen dose effect on immunogenicity in NHP. For anti‐IgE antibody titers, the antigen dose response was absent or small. For example, with Y‐Qb alone, there was no evidence of an antigen dose response from 45 to 900 µg for titers of anti‐IgE Ab. With Y‐Qb and P‐Qb combined, Ab responses were weaker with very low doses of the combination vaccine (2 µg total) compared to higher doses (20 or 200 µg) for at least some time points. An antigen dose effect was also not evident for antibody avidity. Similarly, in mice a weak dose response was seen with mY‐Qb/mP‐Qb combination at lower antigen dose levels 36.

All vaccine formulations were adjuvanted since earlier studies in mice and dogs showed very poor immunogenicity for antigen conjugates alone (Huber et al. & Hedlund et al.; manuscripts in preparation). Most groups received both alum and CpG, but results with alum alone were not inferior to those with alum/CpG except at the lowest antigen dose, indicating a lack of CpG effect once a certain dose of antigen is used. It has been well‐established with a wide variety of antigens, but not including Qb‐VLP conjugates, that alum/CpG significantly enhances antibody responses over alum alone, including in humans studies 50, 55, 56, 57. However, the general lack of adjuvant effect of CpG with Qb‐VLP conjugates was not unexpected due to overlapping adjuvant mechanism; the Qb‐VLP contains RNA that can activate immune cells via Toll‐like receptor (TLR) 7 and TLR8 which use the same MyD88‐dependent pathways as CpG, which binds to TLR9 58. We have also previously demonstrated a lack of synergy between R848 (TLR7 agonist in mice) and CpG as adjuvants with hepatitis B surface antigen in mice 59. Downregulation of innate immune responses following in vitro stimulation of human plasmacytoid dendritic cells and B lymphocytes by multiple closely related TLRs (TLR3, 7, 8, and 9) has been reported 60, 61. This is likely an evolutionary adaptation by the host to prevent overstimulation of the innate immune response.

Some animals had pre‐existing anti‐IgE antibodies, which did not appear to impact the response to the vaccine. The presence of auto anti‐IgE antibodies is well‐established in humans, and have been shown to cause modest (<50%) inhibition of in vitro IgE binding to high affinity receptors using a FcϵRI‐expressing cell‐line, as well as to low affinity CD23 receptors on B cells, suggesting possible anti‐allergic and pro‐allergic effects, respectively 62, 63. Vaccine‐induced anti‐IgE antibodies should augment those binding to FcϵRI, leading to a better anti‐allergic effect.

The vaccine‐induced anti‐IgE antibodies resulted in significant lowering of serum IgE levels in many of the animals, with some animals achieving as much as 80% lower IgE compared to baseline. Despite the lack of antigen dose effect on antibody titer and avidity, there was a clear trend for better functional responses (IgE lowering in blood) with higher antigen doses, suggesting that the assays for titer and avidity are not adequate predictors of anti‐IgE vaccine function. However, despite an increase in the overall antibody titer, addition of P‐Qb with Y‐Qb did not appear to improve the functionality of the antibody over what was seen with Y‐Qb alone at same dose. This may be reflective of the poor quality of anti‐P antibodies; likely due to conformational restrictions of the epitopes within the P peptide. Various studies have illustrated the benefits associated with introducing conformational constraints such as disulphide constrained loops to enhance affinity of peptide vaccines 28, 64. Several such modifications were evaluated for both Y and P peptide conjugates to improve the cross‐reactive anti‐IgE responses induced by the P peptide but no improved cross‐reactivity was found (data not shown).

In clinical studies, adequate dosing of omalizumab has been shown to reduce serum free IgE levels by more than 95% in the majority of patients 17. Baseline levels of IgE seen in the cynomolgus monkeys are higher than typically seen in humans, even atopic humans 65, 66, therefore, it is challenging to compare the degree of reduction of circulating IgE in humans following omalizumab treatment to that seen in NHP following anti‐IgE vaccination. Furthermore, studies have shown that omalizumab has to be given at much higher doses in cynomolgus monkeys than in humans to achieve noticeable levels of free IgE suppression 67.

Predicted free IgE levels in asthmatic patients treated with omalizumab were correlated with reported clinical outcomes and a target level of 14 ng/mL was selected to achieve desired clinical benefit, with clear loss of clinical control above ∼30 ng/mL 68. This is an extremely low level of free IgE and one that may be difficult to achieve with a vaccine. Also, unlike omalizumab, which is delivered as a bolus subcutaneous injection; a vaccine will induce a gradual rise in polyclonal anti‐IgE antibodies with a different kinetics for antibody and IgE‐antibody complex formation to omalizumab. Furthermore, a vaccine‐induced polyclonal anti‐IgE antibody response will also differ in a number of other ways to administering high dose of a single high affinity monoclonal antibody such as the antibody isotypes, avidity, precise specificity, glycosylation patterns, etc. Vaccine‐induced antibodies may also have different functional activities. For example, some anti‐IgE antibodies that can recognize epitopes on membrane bound IgE expressed by IgE class switched B‐cells have been shown to induce tolerance or apoptosis in IgE secreting B‐cells 69, and thereby reduce IgE production. In fact, the murine mimetic of the vaccine described herein also appears to reduce IgE through the loss of IgE‐producing B cells (Fraser et al., manuscript in preparation). In the current study, a number of animals showed sustained reduction in circulating IgE (>50%) compared to baseline at 14 weeks after the second immunization despite waning of antibody titers to levels seen after the priming dose when IgE was not reduced as much. Although we did not directly measure the impact of vaccine‐induced antibodies on membrane IgE expressing B cells, this sustained reduction of circulating IgE may be indicative of loss of IgE production due to elimination of IgE‐producing B cells. In clinical studies with quilizumab (Genentech), a monoclonal Ab targeting the M1’ region of IgE bound to B cells, IgE was reduced only about 25%, presumably due to the elimination of short‐lived but not long‐lived IgE‐producing plasma cells 70.

Although it was beyond the scope of the current study to evaluate therapeutic efficacy of the vaccine‐induced antibodies in monkeys, this was evaluated using mouse and dog mimetics. With mouse prophylactic and therapeutic allergy models and in dog hypersensitive to house dust mites, mimetic vaccines to the human anti‐IgE vaccine showed clear symptomatic benefit despite serum IgE levels being reduced less than 90% 36, 39.

Omalizumab binds with high affinity to free IgE, preventing its binding to the FcϵRI receptor and while free IgE is drastically reduced, total IgE is greatly increased. Such an increase in total IgE was not detected with the vaccine described here in and pre‐clinical data in mice suggest that the anti‐IgE antibodies do not prevent IgE from binding to its receptor, but rather there is an increased clearance rate of IgE with reductions of both free and total IgE 36, 37.

In summary, a novel peptide conjugate anti‐IgE vaccine was tested in NHP and elicited anti‐IgE antibodies that, at least in some animals, could bring about substantial IgE lowering after 3 or 4 vaccine doses. Although the degree of IgE lowering in NHP is less than that seen with omalizumab in humans, the differences in species and potential mechanism of action make it hard to predict how this would translate into humans. Therefore, a decision was made to advance this anti‐IgE vaccine into human clinical testing.

## Conflict of Interest

None declared.
